# Phenotypic Plasticity in *Drosophila* Pigmentation Caused by Temperature Sensitivity of a Chromatin Regulator Network

**DOI:** 10.1371/journal.pgen.0030030

**Published:** 2007-02-16

**Authors:** Jean-Michel Gibert, Frédérique Peronnet, Christian Schlötterer

**Affiliations:** 1 Institut für Tierzucht und Genetik, Veterinärmedizinische Universität Wien, Vienna, Austria; 2 Université Pierre et Marie Curie-Paris, UMR7622-Biologie du Développement, Centre National de la Recherche Scientifique, Paris, France; 3 Institut für Ökologie, Universität Innsbruck, Innsbruck, Austria; Stanford University School of Medicine, United States of America

## Abstract

Phenotypic plasticity is the ability of a genotype to produce contrasting phenotypes in different environments. Although many examples have been described, the responsible mechanisms are poorly understood. In particular, it is not clear how phenotypic plasticity is related to buffering, the maintenance of a constant phenotype against genetic or environmental variation. We investigate here the genetic basis of a particularly well described plastic phenotype: the abdominal pigmentation in female *Drosophila melanogaster.* Cold temperature induces a dark pigmentation, in particular in posterior segments, while higher temperature has the opposite effect. We show that the homeotic gene *Abdominal-B (Abd-B)* has a major role in the plasticity of pigmentation in the abdomen. *Abd-B* plays opposite roles on melanin production through the regulation of several pigmentation enzymes. This makes the control of pigmentation very unstable in the posterior abdomen, and we show that the relative spatio-temporal expression of limiting pigmentation enzymes in this region of the body is thermosensitive. Temperature acts on melanin production by modulating a chromatin regulator network, interacting genetically with the transcription factor *bric-à-brac (bab),* a target of *Abd-B* and *Hsp83,* encoding the chaperone Hsp90. Genetic disruption of this chromatin regulator network increases the effect of temperature and the instability of the pigmentation pattern in the posterior abdomen. Colocalizations on polytene chromosomes suggest that BAB and these chromatin regulators cooperate in the regulation of many targets, including several pigmentation enzymes. We show that they are also involved in sex comb development in males and that genetic destabilization of this network is also strongly modulated by temperature for this phenotype. Thus, we propose that phenotypic plasticity of pigmentation is a side effect reflecting a global impact of temperature on epigenetic mechanisms. Furthermore, the thermosensitivity of this network may be related to the high evolvability of several secondary sexual characters in the genus *Drosophila.*

## Author Summary

The phenotype of an individual is not fully controlled by its genes. Environmental conditions (food, light, temperature, pathogens, etc.) can also contribute to phenotypic variation. This phenomenon is called phenotypic plasticity. We investigate here the genetic basis of the phenotypic plasticity of pigmentation in the fruit fly *Drosophila melanogaster. Drosophila* pigmentation is strongly modulated by temperature, in particular in the posterior abdominal segments of females. The development of these segments is controlled by the homeotic gene *Abdominal-B (Abd-B). Abd-B* sensitizes pigmentation patterning in this region of the body by repressing several crucial pigmentation enzymes. It makes the regulation of their spatio-temporal expression in the posterior abdomen particularly sensitive to temperature variation. We show that temperature modulates the mechanisms regulating the dynamic structure of the chromosomes. Chromosomal domains can be compacted and transcriptionally silent, or opened and transcriptionally active. Temperature interacts with a network of chromatin regulators and affects not only the regulation of pigmentation enzymes but several traits under the control of this network. Thus, we conclude that the phenotypic plasticity of female abdominal pigmentation in *Drosophila* is a visible consequence for a particularly sensitive phenotype, of a general effect of temperature on the regulation of chromosome architecture.

## Introduction

Phenotypic plasticity and buffering are concepts describing the phenotypic outcome of genotype-environment interactions. Phenotypic plasticity is the ability of a given genotype to produce different phenotypes in different environments [1]. It has been the subject of increasing interest as it is involved in adaptation and evolution [1–7]. Buffering, or canalization, is the ability of an organism to maintain a stable phenotype despite genetic variation or environmental fluctuations [8]. Buffering can be challenged by environmental stress, such as extreme temperatures. Thus, the question arises whether the plasticity of a particular phenotype is a specifically targeted reaction of the organism to changes in the environment or whether it is a side effect, reflecting a global process at the level of the transcriptome/proteome, but visible for weakly buffered phenotypes. To answer this question, we investigated the genetic basis of a particularly well described trait subject to phenotypic plasticity: the abdominal pigmentation of female *Drosophila melanogaster,* which strongly depends on the temperature conditions during development [9,10]. In the posterior abdomen, the differences of pigmentation between females grown at 20 °C and 29 °C are comparable to the phenotypic effect of mutations in major structural or developmental regulatory genes. The extreme plasticity of this phenotype makes it a particularly suitable model to dissect the responsible mechanisms. Within the last ten years, key studies have identified structural and developmental regulatory genes playing major roles in abdominal pigmentation patterning [11–16]. Because these studies focused on genetic factors, they were performed under standard temperature conditions [11,13–17]. Following a classical developmental genetics approach, we use mutations in key regulatory or structural genes to destabilize the underlying genetic networks and analyze how they interact with temperature.

## Results

### Spatial Restriction of Pigmentation Plasticity: *Abd-B* Sensitizes Pigmentation Patterning to Temperature in the Posterior Abdomen

**Figure 1:**
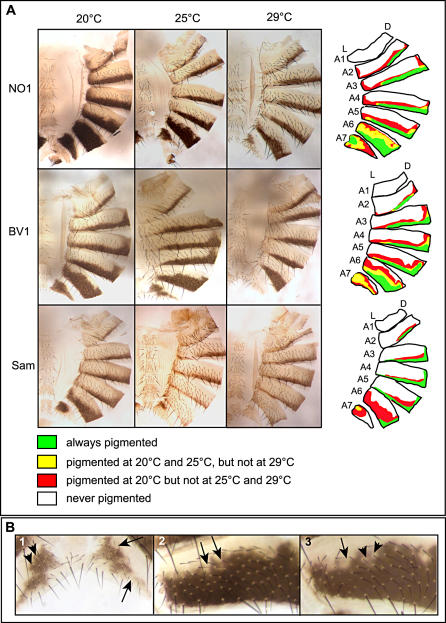
The Pattern of Abdominal Pigmentation Plasticity in Wild-Type Females (A) Abdominal phenotypes of females from the inbred wild-type lines NO1, BV1, and Samarkand grown at 20 °C, 25 °C, and 29 °C. The drawings on the right summarize the plasticity of the different regions of the body according to the color code. A1–A7, abdominal segment number; L, lateral region; D, dorsal region; SAM, Samarkand. (B) The pigmentation in wild-type females shows a variegated pattern. (1) Left and right 7th hemitergites of a BV1 female grown at 25 °C. The pigmentation is not perfectly symmetrical. White patches are visible on one side (arrows) where dark pigmentation is observed on the other side. The dark pigmentation follows the insertion of the small bristles in the inner region of the tergite (arrowheads). (2 and 3) Shows the 6th hemitergites of two NO1 females grown at 25 °C. The pigmentation patterns are very similar but not perfectly identical. The limit between the dark and yellowish regions of the tergite is not smooth but variegated. Yellowish patches (arrows) at the base of some bristles are surrounded by dark pigmentation (3, arrowheads).

Pigmentation is sexually dimorphic in *D. melanogaster.* In males, the abdominal tergites 5 and 6 are black and maintain this pigmentation at all temperatures. In contrast, the posterior abdominal pigmentation in females is highly polymorphic and plastic [18,19]. Figure 1A shows the abdominal pigmentation phenotypes of females from three different wild-type genotypes grown at different temperatures. At a given temperature, the extent of the dark region of the segments in females can differ dramatically between *Drosophila* lines, showing strong genetic basis [18,19]. NO1 and Samarkand are outliers and most lines have a pattern similar to that of BV1, comparable to the patterns described previously through pigmentation score [10]. Differences in plasticity are observed within each segment along the antero-posterior axis [10] and along the dorso-ventral axis (Figure 1A). This is extreme for A7, which can shift from completely black at 20 °C to completely yellowish at 29 °C. In addition, the transition border between the yellowish and the dark region of the tergites is not smooth but variegated (Figure 1B), implying that the control of pigmentation is not robust.

**Figure 2:**
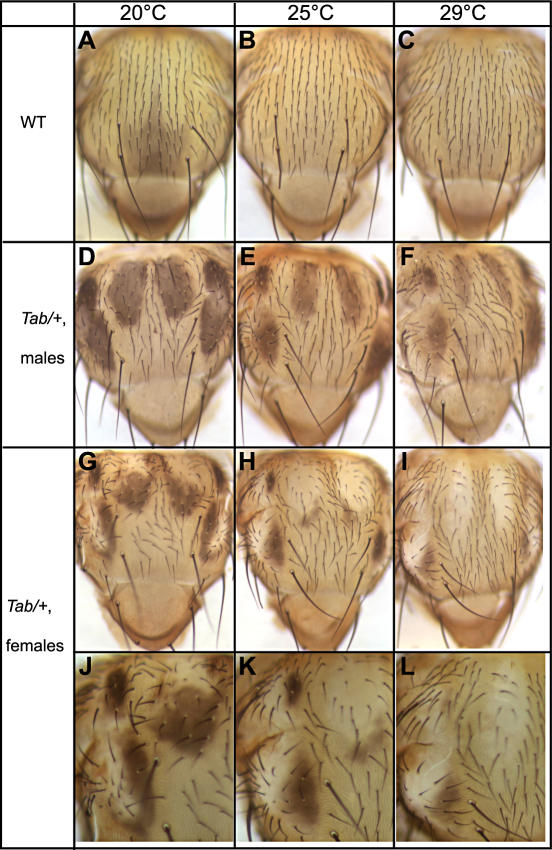
Ectopic Expression of *Abd-B* on the Thorax Is Sufficient to Generate a Sex-Specific Plastic Pigmentation Pattern Effect of temperature on the pigmentation pattern generated by the ectopic thoracic expression of *Abd-B* in the mutant *Transabdominal (Tab)* [17] in males (D–F) and females (G–L). In wild-type flies (A–C), the pigmentation is homogenous in both sexes except for the trident pattern visible at extreme developmental temperature (A). Ectopic expression of *Abd-B* on the thorax is sufficient to generate a highly plastic pigmentation pattern in females (G–L).

The spatial restriction of the phenotypic plasticity of pigmentation suggests the involvement of developmental regulatory genes. The morphology of abdominal tergites A5, A6, and A7 is specified by the posterior homeotic gene *Abdominal-B (Abd-B)* [20]*.* In tergites, *Abd-B* expression is low in A5, intermediate in A6, and high in A7 [21]. Thus, the increasing plasticity observed in the abdomen along the antero-posterior axis [10] perfectly correlates with the expression level of *Abd-B.* We used the *Transabdominal* mutation [17] to test whether ectopic expression of this gene in another body region is sufficient to generate a plastic pigmentation pattern. This mutation is a chromosomal rearrangement that fused the regulatory region of the *stripe* gene to the *Abd-B* locus [17,22]. It induces an ectopic expression of *Abd-B* on the thorax at the flight muscle attachment sites. This phenotype was previously described as sexually dimorphic, inducing melanin production in the whole sites of ectopic expression in males and in only restricted areas in females [17]. The pattern is indeed sexually dimorphic, but it is also extremely plastic (Figure 2). Remarkably, in females, the regions that are not brown at the sites of ectopic *Abd-B* expression show a very strong reduction in the production of yellowish pigments (Figure 2G–2L). This indicates that *Abd-B* plays opposite roles in melanin production. It either increases melanin production or represses the production of all pigments. Furthermore, these two roles are extremely thermosensitive. The increase of melanin production is much higher at low temperature, whereas the decrease in pigment production is much stronger at high temperature. These two roles of *Abd-B* are concomitantly observed within the same spot of ectopic expression, which suggests that they are influenced by other developmental pathways.

**Figure 3:**
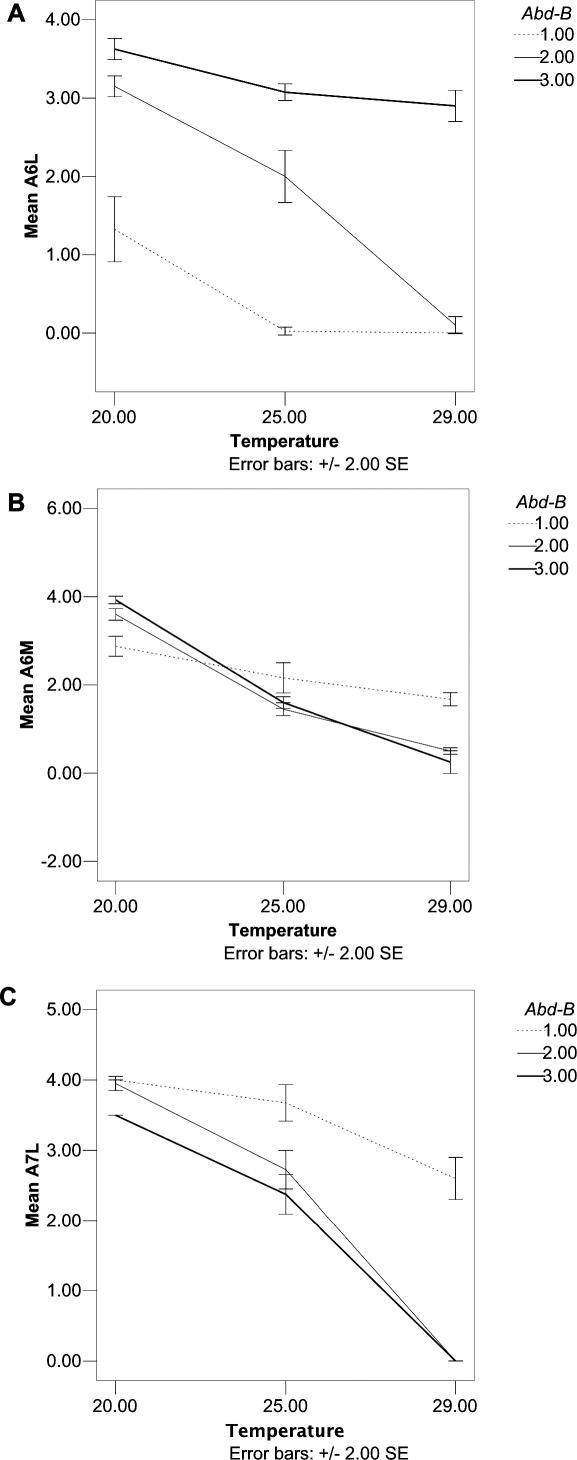
*Abd-B* Interacts with Temperature in the Regulation of Melanin Production Shows the effect of variation of *Abd-B* dosage (1, 2, or 3 copy number) and temperature on melanin production in the lateral region of A6 (A), the median region of A6 (B), and the lateral region of A7 (C).

In order to quantify the effects of *Abd-B* and temperature on pigmentation, we tested how modifications of *Abd-B* expression level interact with temperature in the development of the pigmentation pattern. We varied the copy number of *Abd-B* using a deficiency of *Abd-B (Df(3R)-RS-1–98)* and a duplication of *Abd-B (Dp-P5).* Both mutations are carried in the same stock, which reduces background effects as much as possible. We found that high temperature decreases melanin production in all genotypes, but the effects of *Abd-B* level differed in A6 and A7 and along the dorso-ventral axis within A6 (Figure S1A–S1I). Thus, we quantified the melanin production along the antero-posterior axis in the lateral, median, and dorsal region of A6, in the lateral region of A7, and along the dorso-ventral axis in A7 (Figure S2). Temperature, *Abd-B* as well as the *Abd-B*xtemperature interaction, strongly influenced pigmentation in each of these regions (*p* < 0.001 for all), except the dorsal midline (Table S1). These effects explained a large proportion of the total variation in abdomen pigmentation. The *Abd-B*xtemperature interactions are particularly striking in the lateral regions of A6 and A7 and in the median region of A6 (Figure 3). In line with previous studies [13], *Abd-B* strongly increases melanin production in the lateral region of A6 under all temperature regimes (Figure 3A). The *Abd-B*xtemperature interaction in the lateral region of A6 was mainly attributable to the pronounced reduction in pigmentation at high temperatures when the expression of *Abd-B* is low (Figure 3A). There was also a significant interaction between *Abd-B* and temperature in the lateral region of A7 (Figure 3C). However, in contrast to the lateral region of A6, *Abd-B* represses melanin production in the lateral region of A7. The opposite roles of *Abd-B* on melanin production are best illustrated by the very pronounced *Abd-B*xtemperature interaction in the median region of A6. At low temperature, high *Abd-B* levels increase melanin production, whereas at high temperature they reduce melanin production (Figure 3B).

Based on these data, we conclude that *Abd-B* has two opposite roles on melanin production in females and can either increase or decrease melanin production. This makes the balance between melanin production and repression very unstable in the posterior abdomen, generating phenotypic plasticity in pigmentation. Indeed, this balance is very sensitive to temperature and is most pronounced in A7 showing the highest *Abd-B* level.

### Temperature Modulates the Spatio-Temporal Expression of Limiting Pigmentation Enzymes in the Posterior Abdomen

*Abd-B* is a developmental regulatory gene encoding a homeodomain transcription factor [23]. Its opposite roles on melanin production must be ultimately mediated by pigmentation enzymes. Indeed, pigment precursors move only a few cell diameters; thus, the spatial restriction of some of the enzymes synthesizing them is directly responsible for the pigmentation pattern observed in the adult [14,24,25]. A consensus model of pigment synthesis pathway is discussed in Text S1. Two classes of enzymes can be distinguished. Enzymes of the first class such as the tyrosine hydroxylase (TH) or the dopa decarboxylase (DDC) are required for the production of pigment precursors involved in the synthesis of all pigments. Enzymes of the second class, such as Ebony, Yellow, or Tan, are involved in the switch between the production of yellowish (NBAD sclerotin) or black-brown (dopa-melanin and dopamine-melanin) pigments [14,26].

The strong reduction in the production of all pigment observed in regions expressing *Abd-B* on the thorax of *Tab/+* females suggests that *Abd-B* represses melanin production through the downregulation of one or several enzymes of the first class. The strong production of melanin observed at low temperature in the posterior abdomen and in some of the regions expressing *Abd-B* on the thorax of *Tab/+* females suggests that *Abd-B* also regulates one or several enzymes of the second class. Mutations in genes encoding enzymes of the first class are homozygously lethal but loss-of-function mutations for enzymes of the second class are homozygously viable and can be used to identify the target(s) of *Abd-B* involved in plasticity. We postulated that if phenotypic plasticity of pigmentation is caused by temperature-dependent activity or regulation of a particular pigmentation enzyme, loss-of-function mutations in the gene encoding this enzyme should generate a nonplastic pigmentation phenotype. We used mutations in *yellow (y^1^), tan (t^1^),* and *ebony (e^1^)* (Figure S3)*.* We observed that the plasticity of abdominal pigmentation is still visible in females mutant for *y* (Figure S3D–S3F)*,* which lack black melanin but still have brown melanin*.* In *t* mutants, melanin is produced in males and females in the posterior abdomen at 20 °C, but is strongly reduced at higher temperatures in both sexes (Figure S3G–S3I and S3U). In contrast, *e* flies remain very dark at high temperature and show very limited plasticity of pigmentation (Figure S3J–S3L). Thus, a functional *e* gene is required for the plasticity of pigmentation. In flies mutant for *t,* which antagonizes *e* [15], the role of *e* is magnified. This suggests that the system responsible for plasticity in A5 or A6 also exists partly in males, but that it is normally hidden by the activity of Tan. The production of melanin in *t* mutants requires the repression or the strong downregulation of *e,* as even the gain-of-function of *y* cannot induce melanin production in the presence of Ebony [14]. Thus, the pigmentation pattern of *t* mutants at different temperatures implies that *e* is differentially regulated at different temperatures, relative to the production of melanin precursors, and that a major temperature-induced regulatory switch occurs between 20 °C and 25 °C.

We thus investigated the expression of the genes encoding Ebony and the two enzymes of the first class, *TH* and *ddc.* In order to visualize the expression of these enzymes, we stained pharate adults dissected out of their pupal case. We first investigated the expression pattern of *e, ddc,* and *TH* at 25 °C using *e-LacZ, ddc-LacZ,* and *UAS-LacZ; TH-Gal4* flies, respectively. We observed that *e, ddc,* and *TH* are highly expressed in the pattern of the thoracic trident (Figure S4). The trident is a cryptic pattern fully visible in *e* mutants, *y; e* double-mutants (Figure S4B and S4C) [14], or when flies are grown at extreme temperatures [27] (Figure 2A). A similar pattern was described with an antibody against Ebony [14]. This suggests that the coexpression of *e* with *TH* and *ddc* assures that most of the locally produced pigment precursors are normally converted into yellowish NBAD sclerotin by Ebony. In the absence of Ebony, the excess of dopamine is converted into dopamine- melanin [14]. Thus, the melanin pattern in the absence of *e* is completely correlated to the spatial expression of TH and DDC in the epidermis.

**Figure 4:**
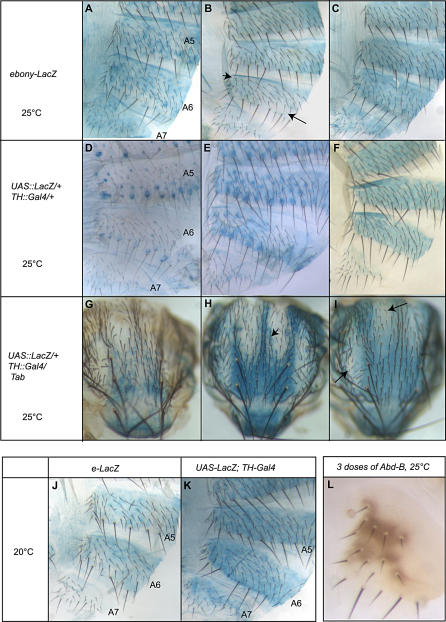
The Expression of Pigmentation Enzymes Is Highly Dynamic and Modulated by Temperature (A–C) Dynamic expression of *ebony-LacZ* at 25 °C in the abdomen of pharate adults. Expression is first visible at the base of bristles around 90 h after puparium formation in (A) and then extends progressively from the anterior region of the segments to all epidermal cells within tergites (B and C). (D–F) Dynamic expression at 25 °C of the tyrosine hydroxylase using *TH-Gal4* and *UAS-LacZ* transgenes. Expression starts earlier than *ebony-LacZ,* at the base of the large bristles on the posterior border of the segments. In (D), the bristles are not mature yet. The expression is then visible at the base of smaller bristles (E), and eventually in all epidermal cells (F). Note that the most posterior tergite, A7, does not show any strong staining. (G–I) Dynamic expression of the tyrosine hydroxylase using *TH-Gal4* and *UAS-LacZ* transgenes in *Tab/+* females at 25 °C at 90 h after puparium formation. Thoraces have an increasing age from left to right, as revealed by the pigmentation at the base of bristles. The abdomen and thoraces shown within the same column do not necessarily have exactly the same age. See text for details. (J) Expression of *ebony-LacZ* in female abdomen just before hatching at 20 °C. No strong expression is visible in A7. (K) Expression of the tyrosine hydroxylase visualized using *TH-Gal4, UAS-LacZ* in female abdomen just before hatching at 20 °C. The expression in A7 is much more visible than at 25 °C. In limiting conditions, such as in females with three doses of *Abd-B* grown at 25 °C, the melanin almost completely disappears from A7 and remains only at the first sites of expression of *TH* associated with bristles (L).

We then looked at the expression of these enzymes in abdominal segments to see how their combined spatio-temporal expression could explain the pigmentation pattern. We observed with the *e-LacZ* transgene an expression similar to that previously reported using an antibody against Ebony [14]. It starts at the base of the bristles around 90 h after puparium formation (Figure 4A) and then becomes progressively uniform in the epidermal cells of the tergites (Figure 4B and 4C). We observed that the epidermal expression starts in the anterior region of the segment, as a weak antero-posterior gradient is first visible (Figure 4B). *e-LacZ* is stronger in the anterior region (Figure 4B, arrowhead) than in the posterior region of the segment (Figure 4B, arrow). We observed that the expression of *ddc* is also very dynamic in the posterior abdomen (unpublished data), but it is even more pronounced for *TH,* which encodes the first and limiting enzyme in the pigment synthesis pathway. Thus, we focused on *TH.* In the abdomen, *TH* expression started at the base of the large bristles on the posterior border of the segments before complete maturation of the bristles (Figure 4D). Expression at the base of more anterior bristles begins later (Figure 4E). Finally, *TH* is later expressed in epidermis of the whole tergites (Figure 4F). In the abdomen, it is expressed along an antero-posterior gradient as the expression starts much later in the more posterior segments (Figure 4D–4F). In particular, no strong expression is visible before hatching in A7 (Figure 4F). Thus, the expression of *TH* is lower in the posterior abdomen where the *Abd-B* level is the highest. We then looked at the expression of *TH* on the thorax of *Tab/+* pharate females. We observed that it is also very dynamic. The expression is first visible at the base of the bristles located in the region of the trident (Figure 4G); then, a uniform expression is visible in the epidermal cells of the trident (Figure 4H). In the regions of ectopic *Abd-B* expression, expression starts at the base of the bristle located close to the teeth of the trident (Figure 4H, arrowhead). Later on, it is visible in the epidermal cells of these regions, but the regions that are devoid of pigments in *Tab/+* flies show a much reduced staining (Figure 4I, arrows). Thus, the most plastic regions, i.e., the posterior abdomen and the regions of *Abd-B* ectopic expression in *Tab/+* females, express *TH* very late. Furthermore, regions of ectopic expression of *Abd-B* devoid of pigments correspond to strongly reduced expression of *TH.* The delayed pigmentation in the posterior abdomen and the loss of *TH* expression in the regions of ectopic expression of *Abd-B* in *Tab/+* females suggest that *Abd-B* represses *TH,* at least indirectly. There is an obvious difference between *e* and *TH* expression at 25 °C in the posterior abdomen. In particular, at 25 °C, *e* is already expressed in A7 before hatching, whereas *TH* is not yet expressed. We assume that *TH* is expressed in A7, which is pigmented, but this expression probably occurs after hatching. This is likely, as the activity of TH was reported to peak 50 min after hatching [28]. This means that when *TH* starts to be expressed, *e* being already expressed, DOPA and dopamine can be used to produce NBAD, the precursor of the yellowish pigment. Because the phenotype of *tan* mutants suggested that a major regulatory switch occurs between 20 °C and 25 °C, we analyzed the expression of *e* and *TH* in females grown at 20 °C. When flies are grown at 20 °C, the expression of *e* just before hatching is much weaker in the posterior abdomen than at 25 °C (Figure 4J). Furthermore, at 20 °C, *TH* expression can be observed very clearly before hatching in A7, but is mainly seen in association with bristles in the inner region of the tergite (Figure 4K).

Thus, *Abd-B* plays opposite roles in melanin production by repressing at least two genes encoding pigmentation enzymes with different roles in melanin production: TH required for the production of all pigments and Ebony required for the production of the yellowish pigment. It makes the expression of these enzymes particularly sensitive to temperature in the posterior abdomen. At low temperature, the stronger repression of *e* and the reduced repression of *TH* correlate with the increased melanin production observed in the posterior abdomen and on the thorax of *Tab+/* females. In contrast, at higher temperature, the strong repression of *TH* and its delayed expression correlate with the reduced pigment production observed in the posterior abdomen and on the thorax of *Tab/+* females. The effect on expression timing is visible on the pigmentation phenotype of the A7 tergite in limiting conditions, for example, in females with three doses of *Abd-B* grown at 25 °C: the melanin remaining is clearly associated with bristles, which mark the first sites of *TH* expression (Figure 4L).

### Temperature Affects a Network of Chromatin Regulators Interacting with the Transcription Factor Bric-à-Brac

**Figure 5:**
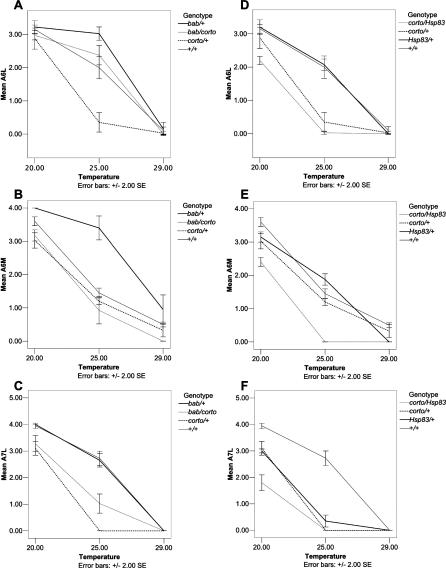
Temperature Interacts with a Network of Regulatory Factors and the Chaperone Hsp90 for the Regulation of Melanin Production Shows the effect of variation of temperature and the alleles *bab^AR07^* and *corto^420^* (A–C) and *corto^420^* and *Hsp83^e6D^* (D–F) on melanin production in the lateral region of A6 (A and D), the median region of A6 (B and E), and the lateral region of A7 (C and F).

**Figure 6:**
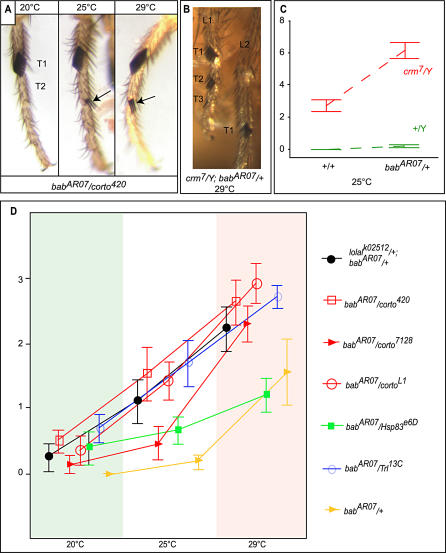
Chromatin Regulators, Temperature, and Sex Comb Development The sex comb is a structure made of modified bristles located on the first tarsal segment of the first leg in males. (A) Sex comb phenotype of representative *bab^AR07^/corto^420^* males grown at 20 °C, 25 °C, and 29 °C. The normal sex comb is located on the first leg in the first tarsal segment (T1). Ectopic sex comb composed of two and three teeth, respectively, can be seen on the second tarsal segment (T2) in the flies grown at 25 and 29 °C (arrows). (B) Sex comb phenotype of *crm^7^/Y; bab^AR07^/+* of a fly grown at 29 °C. An ectopic sex comb caused by *crm^7^* alone is visible on the first tarsal segment (T1) of the second leg (L2), a typical *PcG* phenotype as previously reported [35]. A strong synergistic interaction between *bab* and *crm* is revealed by ectopic sex comb teeth, not only on the second (T2) but also on the third (T3) tarsal segment of the first leg (L1). Note that the legs are shortened and that the second tarsal segment is inflated, affecting the ectopic sex comb shape. (C) Mean and standard errors of sex comb teeth on the second tarsal segment of the first leg in wild-type (green), *bab^AR07^/+*, *crm^7^/Y and crm^7^/Y; bab^AR07^/+* (red) males grown at 25 °C. (D) Mean and standard errors of sex comb teeth on the second tarsal segment in *bab^AR07^/+* single heterozygotes and in combinations with the other mutations indicated on the right grown at 20 °C, 25 °C, and 29 °C.

How could temperature influence these opposite roles of *Abd-B* on pigmentation? *Abd-B* was previously shown to induce melanin production both via the repression of the transcription factor *bric-à-brac (bab)* and independently of *bab* [13]. *bab,* which was shown to repress melanin production, is strongly repressed in males by *Abd-B* in A5 and A6 [13]. This leads to the melanic pigmentation observed in the posterior abdomen [13]. *bab* is activated by the female-specific isoform of *doublesex* (dsxF), which compensates for the repression of *bab* by *Abd-B,* and thus reduces the amount of melanin produced in this part of the abdomen compared to males [13]. In female A7, *bab* is not repressed by *Abd-B* [13]. In order to analyze potential interactions between *bab* and environmental temperature, we used the *bab^AR07^* mutation that completely abolishes the expression of the two paralogs *bab1* and *bab2* and induces a well characterized haplo-insufficient melanic phenotype [13,18,29]. We observed that this phenotype is fully visible at 25 °C in A6, but is less obvious at other temperatures compared to wild-type (Figure S1J–S1L). Multivariate analysis of the effect of *bab* and temperature on melanin production (Table S2) revealed a very strong effect of *bab* and *bab*xtemperature interaction in the lateral and median region of A6 and along the dorso-ventral axis of A7 (*p* < 0.001 for all, Figure 5A and 5B; Table S2). No significant effect was observed in the lateral region of A7 (Figure 5C). Thus, BAB level is less limiting in wild-type flies in A7, where *bab* is not repressed by *Abd-B,* than in A6. The role of *bab* on melanin production has been described previously [13,29], but these experiments did not reveal whether *bab* acts mainly by regulating pigmentation enzymes of the first or the second class. To identify the main targets of *bab,* we overexpressed *bab1* in the dorsal domain using the *pannier* driver as previously described [13], but in an *e* or in a *y* background (Figure 6A and 6B). We observed that the production of both melanin and yellowish NBAD sclerotin is strongly repressed by the overexpression of *bab1* (Figure S5A and S5B, arrows). It suggests that *bab* represses an enzyme of the first class. BAB has been reported to physically interact with products of the *Broad-Complex* [30], a direct regulator of *ddc* in pharate adults [31]. We used a *ddc-lacZ* transgene and observed that *ddc-lacZ* is downregulated in the dorsal domain of flies overexpressing *bab1* (Figure S5C, compared to Figure S5D, arrows). Thus, the effect of *bab* on melanin production is mediated at least through the repression of *ddc.*

Interestingly, temperature was shown previously to modulate the effect of *bab* loss-of-function on another phenotype: the presence of an ectopic sex comb observed in males on the second tarsal segment of the first leg [32]. The sex comb is a structure made with modified bristles present on the first tarsal segment in Drosophila melanogaster males. Ectopic sex comb are extremely informative phenotypes frequently used to identify and quantify particular genetic interactions. The sex comb phenotype of *bab* mutants (distal sex comb) is observed also in some chromatin regulator mutants of the *Polycomb* group *(PcG)* and *Enhancer of Trithorax and Polycomb* group *(ETP)* [33–36]. The Polycomb group (PcG) and the antagonizing Trithorax-group (TrxG) proteins were identified through their role in the regulation of homeotic genes *(Hox)* [37], but it is now clear that they regulate hundreds of genes [38,39]. The PcG are involved in silencing of *Hox* genes, whereas the TrxG are involved in their activation. A third class of chromatin regulators has been described, the Enhancers of trithorax and Polycomb (ETP), required for both TrxG and PcG normal functions [40]. Most *PcG* mutants induce the formation of ectopic sex combs on the first tarsal segment of the second and third legs caused by ectopic expression of the homeotic gene *Sex-comb reduced (Scr)* [37,41]. However, ectopic distal sex combs are observed in mutants of only a few *PcG* or *ETP* genes [34–36]. This suggests that these different ectopic sex comb phenotypes correspond to the disruption of two distinct processes, and that *bab* and a subset of chromatin regulators are required for the repression of the distal sex comb. Other data suggest that these genetic interactions probably correspond to physical interactions between BAB and chromatin regulators. The *bab* locus encodes two closely related transcription factors with a BTB/POZ domain [29]. This interaction domain is present in many chromatin regulators [42] or transcription factors recruiting chromatin regulators [43]. In particular, BAB has been reported to bind to Batman/LolaL, which is part of PcG and TrxG complexes [42]. Interestingly, the activity of chromatin regulators such as members of the Trithorax/Polycomb system are known to be temperature-sensitive [38,44]. Silencing by PcG through characterized regulatory sequences known as Polycomb Response Elements was shown to be stronger at high temperature [38,44,45]. Thus, we hypothesized that the modulation of *bab* activity by temperature could take place via an effect of temperature on a network of chromatin regulators interacting with BAB.

We used the sex comb phenotype to test for genetic interactions between *bab,* genes encoding ETP or PcG, and temperature. We observed strong genetic interactions between *bab* and *corto, cramped (crm), batman/lolal,* and *Trithorax-like (Trl)* that encodes GAGA (Figure 6). Temperature strongly enhances the sex comb phenotype of *crm^7^/Y; bab^AR07^/+* males. At 29 °C, they die in their pupal case with sex comb teeth also visible on the third tarsal segment of the first leg (Figure 6B) in a large fraction of the individuals (6/18 observed legs). This phenotype is not observed at lower temperature or in single mutants. In addition, the second tarsal segment is inflated and shortened, which reduces the size of the ectopic sex comb and makes quantification impossible. Therefore, we quantified the *crm-bab* interaction only at 25 °C using the number of teeth in the ectopic sex comb on the second tarsal segment of the first leg (Figure 6C). Wild-type flies have no sex comb teeth on the second tarsal segment (0 ± 0, *n* = 16). The *crm^7^/Y; bab^AR07^*/+ males have many more teeth (6.18 ± 0.25, *n* = 22) than *crm^7^*/Y hemizygotes (2.75 ± 0.18, *n* = 12) and *bab^AR07^* heterozygotes (0.18 ± 0.05, *n* = 36). This strong genetic interaction is shown in Figure 6C. We also quantified the effect of temperature on the interactions between *bab* and other chromatin regulators (Figure 6A and 6D, Tables S3 and S4). The single heterozygote mutants for *corto, ban,* or *Trl* do not show ectopic sex combs. We analyzed how these mutations modify the *bab^AR07^/+* phenotype. The genotype and the temperature accounted for 71% of the variance (Table S4). Temperature had a strong effect and increased sex comb teeth number across all genotypes. All heterozygote double mutants were significantly different from *bab^AR07^/+* (Tukey post hoc test, *p* < 0.001), which shows that the effect of *bab* mutation is strongly enhanced by mutations in *corto, ban/lolal,* or *Trl.* We tested three *corto* alleles. All alleles showed a similar trend, but the effects were stronger for *corto^420^* and *corto^L1^.* In addition, there was a significant genotype/temperature interaction (Table S4) visible in the curves corresponding to *bab^AR07^* single mutants and double heterozygotes (Figure 6D).

Given these strong effects, we analyzed the effect of mutations in chromatin regulators on the abdominal pigmentation of *bab^AR07^* heterozygote females. All the mutants we looked at had been induced in different backgrounds, so it was not possible to clearly differentiate the effect of the mutation itself from the background. Balancers are frequently used as control when the mutant stock is out-crossed to a wild-type line. However, most of the balancer chromosomes from the mutant stocks carry mutations in pigmentation genes, which is particularly inadequate in our case. Thus, we focused on genes for which we observed very strong phenotypes and interactions, and/or for which we could test different alleles. We observed very strong effects for the three *corto* alleles. They dominantly induce a reduced pigmentation in A7 at 25 °C. This is extreme for *corto^420^* and *corto^L1^* (Figures S6 and S7) and weaker for *corto^07128^* (Figure S7E). We tested how they would modify the haplo-insufficient pigmentation seen in *bab^AR07^* females. We observed a strong temperature-sensitive effect on pigmentation in *bab^AR07^*/*corto^420^* in A6 with a completely black phenotype at 20 °C, a strong variegation at 25 °C, and a completely white pigmentation at 29 °C (except for the dorsal midline) (Figure S7G–S7I). In A7, the pigmentation was very weak at 25 °C. A similar effect was observed with *corto^L1^* (Figure S8) and a visible but weaker effect with *corto^7128^* (Figure S8). Quantification of melanin production revealed very strong effect for *corto^420^* and strong interactions between *corto* and temperature and between *bab, corto,* and temperature (Figure 5A–5C, Table S1). In particular, whereas reducing *bab* level by half has no significant effect on melanin production in the lateral region of A7, it interacts very strongly with *corto* for this phenotype (Figure 5C, Table S1). In addition, in the median region of A6, *bab^AR07^/corto^420^* females produce less melanin than wild-type or single heterozygous females, whereas reducing *bab* level alone has the opposite effect (Figure 5B). This corresponds to the variegated phenotype observed at 25 °C (Figure S7H) and shows that *bab* and *corto* work together to increase melanin production in this region of A6. The females homozygous for the *crm^7^* allele of the *PcG* gene *crm* show an absence of melanin in A7 and a very reduced and variegated pigmentation in A6 (Figure S8A). This is not observed in their heterozygote siblings *crm^7^/FM7c* (Figure S8B) or when out-crossed to a wild-type stock (Figure S8C). We also quantified how heterozygosity for *crm^7^* would modify the pigmentation phenotype of *bab^AR07^/+* females. Except for the dorsal midline, we observed strong effect of *crm* and interaction between *crm* and *bab,* between *crm* and temperature, and between *crm, bab,* and temperature (Table S1).

**Figure 7:**
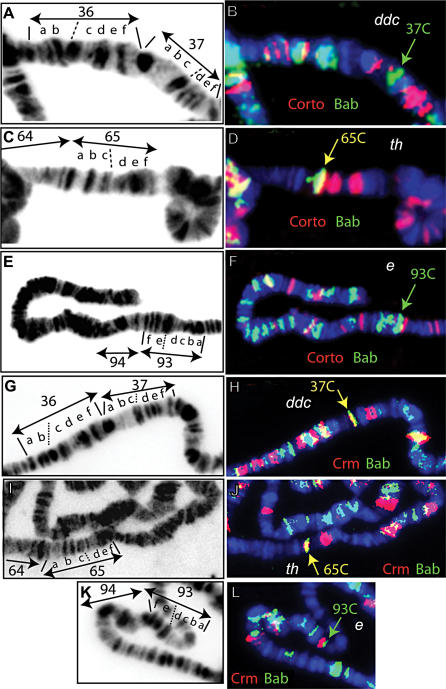
BAB Colocalizes with Chromatin Regulators on Polytene Chromosomes Double immunostaining of polytene chromosomes with antibodies against BAB (green; B, D, F, H, J, and L), Corto (red; B, D, and F) and CRM (red; H, J, and L) in the cytological region of *ddc* (37C; A, B, G, and H), *TH* (65C; C, D, I, and J), and *e* (93C; E, F, K, and L). Colocalizations are indicated by yellow arrows. DAPI staining (blue) allows the visualization of euchromatic (lightly stained) and heterochromatic (densely stained) regions. Inverted black and white pictures of the DAPI staining are shown on the left to identify the cytological regions. Note the staining for BAB in the region of all three enzymes (B, D, F, H, J, and L), the colocalizations in the *TH* region with both Corto (D) and CRM (J), and in the *ddc* region with CRM (H).

The genetic interactions between *bab* and chromatin regulators for abdominal pigmentation and sex comb development, and previously reported physical interaction between BAB and the ETP Batman/Lolal [42], suggest that BAB and chromatin regulators cooperate in the regulation of particular targets. In order to test this hypothesis, we used antibodies against BAB, CRM, and Corto to localize their products on salivary gland polytene chromosomes. We observed many colocalizations on polytene chromosomes of BAB with Corto and BAB with CRM (unpublished data). In particular, we observed clear staining for BAB, Corto, and CRM in the cytological region corresponding to the locus of *TH* (65C) (Figure 7). BAB and CRM colocalized in the cytological region of the *ddc* (37C) (Figure 7G and 7H). We detected BAB alone in the cytological region of *e* (93C) (Figure 7E, 7F, 7K, and 7L).

### This Chromatin Regulator Network Interacts Genetically with the Chaperone Hsp90

Chaperones, in particular Hsp90, have been shown to buffer against the effect of cryptic genetic variation and environmental stress, in particular against high temperature [46,47]. Recent studies revealed a link between the chaperones and chromatin regulators [48–50], which suggested that the effect of temperature on chromatin regulators might be partly mediated by chaperones. We tested this hypothesis using two different alleles of *Hsp83,* the gene encoding Hsp90. In females, the allele *Hsp83^e6D^* dominantly induced a very low pigmentation in A7 at 25 °C (Figure S6Q), not observed with the allele *Hsp83^e6A^* (unpublished data), which had a weaker *TrxG*-like effect than *Hsp83^e6A^* [48].

We tested the effect on abdominal pigmentation of the two *Hsp83* alleles in *bab^AR07^* heterozygous females. We observed that *Hsp83^e6D^* (Figure S6T and S6U), but not *Hsp83^e6A^* (unpublished data), strongly reduced the pigmentation phenotype of *bab^AR07^/+* at 25 and 29 °C. Quantification of melanin production revealed strong effect of *Hsp83^e6D^* and a strong interaction with temperature (Table S1). In contrast, interactions between *Hsp83^e6D^* and *bab* were only significant in the median region of A6. Significant interactions were observed between *bab, Hsp83^e6D^,* and temperature in the median region of A6 and along the dorso-ventral axis of A7 (Table S1). We also quantified the effect of *Hsp83^e6D^* on *bab^AR07^* heterozygote sex comb phenotype and found that it increased the number of teeth in the ectopic sex comb at 20 °C and 25 °C, but slightly decreases it at 29 °C (Figure 6D, Table S4). Because of the similarity of effects of *Hsp83* and *corto* on the pigmentation phenotypes, we tested for potential genetic interactions between these two genes. We observed extragenic noncomplementation (lethality) between *Hsp83^e6D^* and *corto^L1^* (observed when crossed in both directions), whereas *corto^L1^* is viable with *Hsp83^e6A^.* The *Hsp83^e6D^/corto^420^* and *Hsp83^e6D^/corto^07128^* genotypes were viable, but strongly enhanced the loss of pigmentation observed in *Hsp83^e6D^/+*, *corto^420^/+*, or *corto^7128^*/+ females at 25 °C (Figures S6V–S6X and S7P–S7R). Quantification of melanin production revealed very strong interactions between *Hsp83* and *corto* and between *Hsp83, corto,* and temperature (Figure 5D–5F; Table S1). In particular, the double heterozygote *Hsp83^e6D^/corto^420^* females have an extremely reduced melanin production (Figure 5D–5F) and are the only genotype we analyzed where the pigmentation in A6 at the dorsal midline is affected at 25 and 29 °C. Furthermore, the *Hsp83^e6D^* allele (Figure S9) also dominantly induced pigmentation defects in males in the A5 segment observed at 25 °C and 29 °C, but not at 20 °C (Figure S9B and S9C). Most importantly, *corto* alleles strongly increased the pigmentation defects observed in *Hsp83^e6D^*/+ males at 25 °C and 29 °C (Figure S9D–S9I). Loss of pigmentation was visible in A6 with both alleles. In contrast, *corto^420^/+* and *corto^07128^/+* males had a normal pigmentation in A5 and A6 at all tested temperatures (unpublished data). We observed a similar phenotype in *Hsp83^e6D^/bab^AR07^* males (Figure S9J–S9L). This suggests that *Hsp83, bab,* and *corto* work tightly together to control abdominal pigmentation in both males and females and are much more required at 25 °C and 29 °C than at 20 °C.

## Discussion

Buffering or canalization describes the ability of individuals of a given species to show a constant phenotype despite genetic variations or environmental fluctuations. Phenotypic plasticity could therefore be interpreted as a weaker buffering of some phenotypes. Chaperones, and in particular Hsp90, have been identified as components of this buffering system and are thought to become limiting under stressful conditions such as high temperature [46,48]. The chaperone Hsp90 was proposed to act as a general evolutionary capacitor by releasing the effect of cryptic genetic variation under stressful environment [46]. However, more recent studies have revealed that the influence of Hsp90 on the variation of particular traits was very limited [51]. This suggests that the ability of chaperones, and Hsp90 in particular, to buffer phenotypic variation is not so general, but might rely on very specific interactions more tightly involved in particular phenotypes. Recent studies revealed a link between chaperones and chromatin regulators [48–50]. In particular, Hsp90 and several TrxG chromatin regulators were shown to buffer the same phenotype [48]. These studies on buffering in flies were based on the penetrance of deleterious phenotypes caused by cryptic genetic variation or known introduced mutations [46,48]. We found that mild modulation of a similar system by environmental temperature is involved in phenotypic plasticity of abdominal pigmentation. The chromatin regulator network we found to be sensitive to environmental temperature interacts genetically with the chaperone Hsp90 and the transcription factor BAB. It contains the *PcG* gene *crm* and the *ETP* gene *corto.* We observed very strong genetic interactions between *corto* and *Hsp83,* the gene encoding Hsp90. In particular, we observed extragenic noncomplementation between *Hsp83^e6D^* and *corto^L1^,* whereas the viable trans-heterozygote combinations with the two other *corto* alleles induce strong reduction in melanin production in both sexes. This suggests that Hsp90 and Corto are involved in a common process. Hsp90 has been shown to physically interact with histones, to induce chromatin condensation, and to interact with topoisomerase II, which plays a crucial role in chromosome condensation [52,53]. Interestingly, *corto* is also required for the normal condensation of chromosomes during mitosis [33]. Therefore, the interactions between *corto* and *hsp83* in gene regulation might be linked to a general role and common involvement of these genes in chromatin condensation.

**Figure 8:**
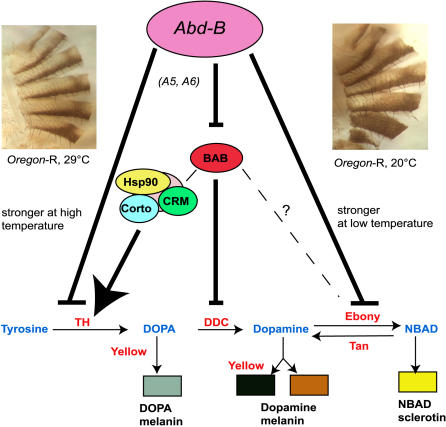
Schematic Representation of the Gene Network Involved in Phenotypic Plasticity of Abdominal Pigmentation One major role of the phenotypic plasticity of abdominal pigmentation is played by *Abdominal-B (Abd-B). Abd-B* represses *ebony (e)* and *tyrosine hydroxylase (TH),* not necessarily directly. The first role is stronger at low temperature and the second role is stronger at high temperature. This correlates with the increase in melanin production at low temperature and the decrease of all pigments at high temperature in the posterior abdomen. *Abd-B* is known to increase pigmentation in A5 and A6 by repressing *bab* [13]. Hsp90, BAB, and chromatin regulators, such as Corto and Cramped (CRM), are particularly limiting at intermediate and high temperature for the expression of *TH*. Thus, at high temperature they do not counteract as strongly as at low temperature the repression of *TH* by *Abd-B*. This reduces melanin production in the posterior abdomen. Colocalizations on polytene chromosomes suggest that they directly regulate *TH.* Some of them may also regulate together the expression of other enzymes, as suggested by the detection of BAB and CRM in the cytological region containing the locus of the *ddc.* Indeed, we showed that BAB represses *ddc,* at least indirectly. Furthermore, the detection of BAB in the cytological region containing *ebony* suggests that it might be also involved in its regulation. The pigment synthesis pathway represented here is a consensus pathway between the models proposed by several authors (see Text S1 for details).

The pigmentation in the posterior abdomen is particularly sensitive to environmental temperature because it is sensitized by the input of the homeotic gene *Abd-B. Abd-B* plays opposite roles in melanin production. The positive role of *Abd-B* in melanin production in females has already been described [13]. It is linked to the establishment of the sexually dimorphic pattern of pigmentation, and, for this role, *Abd-B* works antagonistically with *bab* by repressing it in A6 and A5 [13]. The role of *Abd-B* in repressing melanin production has not been described previously. It is very strong in A7 and is probably linked to the very peculiar morphology of this segment in females. In A7, *bab* is not repressed by *Abd-B,* and both genes work together not only to repress melanin production, but also to control some aspects of the particular development of this segment such as the absence of fusion of the tergites at the dorsal midline [29,54]. *Abd-B* plays these opposite roles in melanin production by repressing several pigmentation enzymes such as *TH* and Ebony. These enzymes start to be expressed at the end of pupal development [14,28]. Modulation of the regulation of these enzymes by temperature induces a local difference in their relative timing of expression in the abdominal epidermis. The effect is particularly visible in A7, which exhibits the highest *Abd-B* level. Studies in *Drosophila* wing have shown that pigment precursors can also be provided by the hemolymph [55]. Hence, it is possible that a change in the general level of pigment precursors in the hemolymph might contribute partly to the phenotypic plasticity of abdominal pigmentation. However, the diffusion of pigment precursors from the hemolymph does not seem to play an important role in the pigmentation of the body: recent studies in lepidopterans showed that the local production of DOPA and dopamine by TH and DDC in the epidermis are major components of the pigmentation pattern [56]. In *Drosophila* abdomen, epidermal clones of cells mutant for *TH* or *DDC* are albino [55], which shows that pigment precursors potentially available in the hemolymph cannot contribute significantly to pigment production in the body epidermis. In addition, in the thorax, the pigmentation patterns visible in *e* and *y, e* mutants perfectly correlate with the epidermal expression of *TH* and *DDC,* the enzymes providing pigment precursors (Figure S4). Furthermore, we clearly show that the spatial restriction of plasticity is strongly conditioned by *Abdominal-B* expression and the repression of pigment precursor production in the thoracic epidermis in *Transabdominal* mutants (Figure 2). Thus, we conclude that the modulation of the relative temporal expression of *TH* and *ebony* by temperature in the epidermis of the posterior abdomen is responsible for the phenotypic plasticity of female abdominal pigmentation. Mutations in *corto, crm, hsp83,* and *bab* enhance the effect of temperature on melanin production in the posterior abdomen. The colocalization of *bab, corto,* and *crm* at the locus containing *TH* in polytene chromosomes suggest that they might all cooperate in the direct regulation of this pigmentation enzyme, and that they might counteract the effect of temperature and *Abd-B* on *TH* expression. Their mutations enhance the repression of *TH* by *Abd-B* and high temperature, which explains why it has a particularly strong effect in A6 and A7, at 25 °C and 29 °C. We therefore propose the model presented in Figure 8 to explain some aspects of the pigmentation pattern plasticity in the posterior abdomen.

It does not exclude that temperature also modifies the expression of other genes. This is likely as the PcG/TrxG have hundreds of targets [38,39], and the thermosensitivity of the PcG/TrxG system is a general phenomenon observed with PRE from several different genes [38,44]. It is possible that other genes (developmental regulators or structural genes) are also modulated by temperature and contribute to the phenotypic plasticity of pigmentation. We observed, indeed, many colocalizations of Corto and BAB, and Corto and CRM, suggesting that this particular network of chromatin regulators regulate many targets. We demonstrate that this network is involved in at least two different phenotypes, abdominal pigmentation in females and sex comb development in males, both showing high temperature sensitivity. Thus, we propose that the plasticity of *Drosophila* pigmentation is a visible side effect, at particularly sensitive loci, of a process affecting the whole genome through alteration of epigenetic mechanisms.

Interestingly, abdominal pigmentation and morphology of the sex comb along the proximo-distal axis of the first leg evolve very rapidly in the *Drosophila* genus [57–59]. Remarkably, we found that these two morphological traits are under the control of a common thermosensitive network including the transcription factor *bab,* chromatin regulators, and the chaperone Hsp90. This suggests that the thermosensitivity of this particular regulatory network might be linked to the high evolvability of several secondary sexual characters in the genus *Drosophila.* Our results corroborate other studies, which have shown that the plasticity of specific traits is correlated to their evolvability [60].

## Materials and Methods

### Origin and maintenance of fly stocks, crosses.

Most of the fly stocks used in this study were provided by the Bloomington *Drosophila* Stock Center (http://flystocks.bio.indiana.edu). The following ones were kindly sent to us by various researchers: *bab^AR07^*and *UAS-bab1* (Jean-Louis Couderc), *crm^7^* (Neel Randsholt), *Df(3R)RS-1–98/Dp-P5* (Artyom Kopp), *ebony-LacZ* (Bernard Hovemann), and *Pc^3^ Tab* (Ian Duncan)*.* The *Tab* mutation is associated with the *Pc^3^* inversion in the stock we used, but the pigmentation phenotype visible on the thorax has been shown to be caused by the ectopic expression of *Abd-B* [17]. Flies were grown on standard agar-corn medium. Standard balancer chromosomes were used. For the effect of temperature, crosses were kept at 25 °C and tubes transferred after 2 d to the desired temperature. Oregon*-*R was used as a wild-type stock to outcross mutant stocks. All fly stocks are described in Flybase (http://flybase.bio.indiana.edu).

The interaction between *bab,* chromatin regulators, and chaperones was analyzed using sex comb phenotypes (except for the *bab/crm* interaction) and female abdominal pigmentation in the progeny of crosses between females Oregon-R or *bab^AR07^/+* and males carrying the mutation to be tested, or wild-type Oregon-R males. The interactions between *crm* (located on the X chromosome) and *bab* on sex comb was analyzed in the male progeny of crosses between *crm^7^/FM7c* females and males *bab^AR07^/TM6b,* and compared to the effect of these mutations alone when crossed to Oregon-R. The effect and interaction with *corto* and *bab* of the *hsp83^e6D^* allele on male abdominal pigmentation was observed and analyzed in the male progeny of crosses made with females *hsp83^e6D^/TM6b* and wild-type males or carrying the mutation to test.

### Abdominal and thoracic cuticle preparation.

Flies were fixed in 75% ethanol 3 d after hatching to allow proper pigmentation of the cuticle to develop. Abdominal cuticles were cut on one side of the dorsal midline, cleaned, and mounted in Euparal (Roth). At least 15 flies were observed for each genotype/temperature condition, except the *crm^7^* homozygote female escapers (Figure S3A) for which we had only five individuals. Thoraces were dissected in 75% ethanol, fixed 5 min in 100% ethanol, and mounted in Euparal.

### *LacZ* staining.

Flies were dissected out of their pupal case, fixed, and stained without further dissection with X-Gal according to previously described protocols for pharate abdomen [61,62]. Because developmental time is conditioned by temperature [63], we staged pharate adults according to progression of eye color, bristle and wing melanization, meconium appearance, and ability of the fly to walk prematurely when dissected out of the pupal case [64]. Stainings were performed overnight at 37 °C for *e-LacZ* and *ddc-LacZ,* or 2 h for *TH-GAL4; UAS-LacZ* genotypes. Thoraces or abdomen were dissected after staining in PBS. This allowed us to make sure that absence of staining was not caused by tissue disruption during dissection prior to staining. Tissues were then dehydrated 5 min in 75% ethanol, 5 min in 100% ethanol, and mounted in Euparal.

### Polytene chromosomes.

Immunostaining of polytene chromosomes was performed as described by Cavalli (http://www.igh.cnrs.fr/equip/cavalli/link.labgoodies.html) on larvae of the *w^1118^* genotype. The rabbit anti-CRM [35], rat anti-BAB2 [30], and rabbit anti-Corto [65] antibodies were used respectively, at 1:50, 1:200, and 1:25 dilutions.

### Statistical analysis of sex comb teeth number and pigmentation phenotypes.

All statistical analyses were performed using the software SPSS.10.0 or SPSS.13 [66]. We scored sex comb teeth number in the ectopic sex comb on the second tarsal segment. The number of teeth on the left and right legs of individuals was highly correlated for the *bab-crm* interaction (*rs* = 0.892, *p* < 0.001), as well as for *bab* and other chromatin regulator interactions across temperatures (*rs* = 0.667, *p* < 0.001). Therefore, we averaged the number of teeth of the left and right leg for all subsequent analysis. We analyzed the effect of mutations in *bab* and chromatin regulators on sex comb teeth number at three temperatures using a parametric two-way ANOVA. Log transformed data (ln + 1) were not exactly normally distributed; however, the residuals of the analysis did not deviate from normality (Kolmogorov-Smirnov test, *p* > 0.05).

We observed that the different genetic combinations affected melanin production differently in abdominal segments A6 and A7. Furthermore, within segments the effects differed along the dorso-ventral axis. Thus, we scored the proportion of melanin visible along the antero-posterio axis at the dorsal midline (A6D), on both sides in the lateral region of A6 (A6L1, A6L2), and in the median region of A6 (A6M1, A6M2) (Figure S2). In A7 we scored the proportion of melanin on both sides along the dorso-ventral axis (A7DV1, A7DV2) and the lateral region along the antero-posterior axis (A7L1, A7L2) (Figure S2). Ten individuals were scored for each genotype/temperature combination. Pigmentation scores varied between 0 (no melanin) and 4 (fully black) (Figure S2). Pigmentation scores between the left and right side were highly correlated within all regions (all: *rs* ≥ 0.925, *p* < 0.001). They were averaged in each individual (A6L, A6M, A7L, A7DV). We analyzed these pigmentation data using a multivariate analysis of variance. A6L, A6M, A6D, A7L, and A7DV were used as dependent variables; temperature and genotypes at *Abd-B* (one, two, or three doses), *bab* (one or two doses), *corto* (wild-type or *corto^420^*/+), *Hsp83* (wild-type or *Hsp83^e6D^*/+), and *crm* (wild-type or *crm^7^*/+) as fixed factors. We included all main effects as well as possible interaction terms in the model (Table S1).

The model includes genes interacting with *bab* (encoding putative cofactors). Thus, in this model, although the effect of *bab* and the interaction between *bab* and temperature are highly significant (Table S1), they are also allocated to the interactions between *bab* and other genes, and between *bab,* other genes, and temperature. In order to test more generally the effect of *bab* and its interaction with temperature before dissecting the network (see Results), we also performed a multivariate analysis using the same dependant variables: temperature and genotype at *bab* as fixed factors in a reduced dataset with only wild-type and *bab^AR07^/+* females (Table S2).

## Supporting Information

Text S1 Consensus Model of Pigment Synthesis Pathway (63 KB DOC)

Figure S1 *Abd-B* and *bab* Interact with Temperature for the Regulation of Melanin Production (A–I) Effect of modulation of *Abd-B* copy number on the plasticity of the abdominal pigmentation pattern in females: one dose *(Df(3R)RS-1–98),* two doses (wild-type), and three doses *(Dp-P5).*
(J–L) Effect of the reduction of *bab* expression level on the plasticity of the abdominal pigmentation pattern.
(7.7 MB TIF)

Figure S2 Drawing Illustrating How We Scored the Pigmentation Phenotypes The pattern of melanin is differently affected by genetic factors and temperature in A6 and A7 and along the dorso-ventral axis. Thus, we scored the proportion of melanin in A6 along the antero-posterior axis at the dorsal midline (A6D), on both sides in the lateral region of A6 (A6L1, A6L2), and in the median region of A6 (A6M1, A6M2). In A7, we scored the proportion of melanin on both sides along the antero-posterior axis in the lateral region (A7L1, A7L2) and along the dorso-ventral axis (A7DV1, A7DV2). Pigmentation scores varied from 0 (no melanin) to 4 (fully black).
(363 KB TIF)

Figure S3 Phenotypes of Pigmentation Mutants Grown at 20 °C, 25 °C, and 29 °C (A–L) Female hemitergites of abdominal segments number 4 to 7. (M–X) Male hemitergites of abdominal segments 1 to 6. Orientation: anterior up and dorsal right. (A–C) and (M–O) wild-type. (D–F) and (P–R) *yellow* mutant *y^1^.* (G–I) and (S–U) *tan* mutant *t^1^.* (J–L) and (V–X) *ebony* mutant *e^1^.* Female pigmentation shows a strong plasticity in the posterior abdomen in wild-type (A–C). In comparison, male pigmentation is very stable. However, in some wild-type lines, the fourth abdominal segment in males shows a noticeable plasticity, with increased pigmentation at low temperature (M, arrow). Mutation in *y* does not modify the pattern of plasticity. It is shown that the production of yellowish pigments is reduced in A7 at 29 °C compared to more anterior segments (F, arrow). Mutation in *t* strongly increases the effect of high temperature. Even males lose pigmentation. This is also visible in the anterior region of A5 at 20 °C (S, arrow). The *e* mutation almost completely cancels the effect of high temperature.
(7.1 MB TIF)

Figure S4 Thoracic Pigmentation Phenotypes and Expression of Pigmentation Enzymes Thoracic phenotype of a wild-type (Oregon-R) fly (A), an *e^1^* mutant (B), and a *y^1^; e^1^* double-mutant (C). (D) Thoracic expression of *tyrosine hydroxylase* visualized with *TH-Gal4; UAS-LacZ*. (E) Thoracic expression of *ddc-lacZ*. (F) Thoracic expression of *ebony-LacZ.* All flies were grown at 25 °C. Note that all three enzymes are upregulated in the pattern of the trident visible in *e^1^* mutants and *y^1^, e^1^* double-mutants.
(6.6 MB TIF)

Figure S5 *bab* Represses *ddc*
Shows abdominal phenotype of the ectopic expression of *bab1* in the dorsal domain using the driver *Pannier-Gal4* and *UAS-bab1* in an *ebony* (A) and *yellow* (B) background. Flies were grown at 20 °C because the gain-of-function experiment was lethal at higher temperature. They are thus very pigmented. Pigments disappear in the dorsal domains in both experiments (arrows). (C) Expression of the *ddc-LacZ* at 20 °C in female abdomen. (D) Expression of *ddc-LacZ* at 20 °C in a female overexpressing *bab1* in the dorsal domain. Note the downregulation of *LacZ* in the dorsal domain.
(5.9 MB TIF)

Figure S6 Chromatin Regulators, *bab,* and the Chaperone Hsp90 Interact with Temperature in the Regulation of Melanin Production Abdominal pigmentation phenotypes in females carrying combinations of mutation in the transcription factor *bric-à-brac (bab),* the chromatin regulators *corto* or *cramped (crm),* or *Hsp83,* encoding the chaperone Hsp90, grown at 20 °C, 25 °C, and 29 °C. See text for details.
(6.7 MB TIF)

Figure S7 Interactions with Other *corto* Alleles Abdominal pigmentation phenotypes in females carrying combinations of mutation in the transcription factor *bric-à-brac (bab),* the chromatin regulator *corto* or *Hsp83,* encoding the chaperone Hsp90, grown at 20 °C, 25 °C, and 29 °C. See text for details.
(8.9 MB TIF)

Figure S8 Effect of the *crm^7^* Allele on Melanin Production in Female Abdomen Abdominal pigmentation phenotypes of a *crm^7^* homozygote female, a *crm^7^/FM7C* sibling heterozygote with the balancer from the stock, and a heterozygote *crm^7^* female obtained in a cross with the wild-type line Oregon-R. Note the strong reduction of melanin production in abdominal segments A6 and A7 in (A).
(3.1 MB TIF)

Figure S9 Abdominal Phenotypes of Males Heterozygote for the *hsp83^e6D^* Allele of the Gene Encoding the Chaperone Hsp90 in an otherwise Wild-Type (A–C), *corto^07128^/+* (D–F), *corto^420^*/+ (G–I), or *bab^AR07^/+* (7–9) Background All genotypes were grown at 20, 25, and 29 °C. Small patches where melanin production is abnormal are indicated in (B and C, arrows). Males heterozygous for *corto^07128^/+*, *corto^420^/+*, and *bab^AR07^/+* have a normal pigmentation in A5 and A6 at all three temperatures (unpublished data).
(5.4 MB TIF)

Table S1 Multivariate Analysis of the Effect of Temperature and Particular Regulatory Genes on Melanin Production We analyzed the lateral region of A6 (A6L), the median region of A6 (A6M), the dorsal midline of A6 (A6D), the lateral region of A7 (A7L), and along the dorso-ventral axis in A7 (A7DV).
(22 KB DOC)

Table S2 Multivariate Analysis of the Effect of Temperature and the Gene *bab* on Melanin Production We analyzed the lateral region of A6 (A6L), the median region of A6 (A6M), the dorsal midline of A6 (A6D), the lateral region of A7 (A7L), and along the dorso-ventral axis in A7 (A7DV).
(22 KB DOC)

Table S3 Effect of Mutations of *bab* and Chromatin Regulators in Males on Sex Comb Teeth Number on the Second Tarsal Segment of the First Leg Mean and standard errors of sex comb teeth number and sample size of individuals scored. Unlike *bab^AR07^,* the other mutations do not induce the formation of ectopic sex comb on the second tarsal segment, so we analyzed how they modify the *bab^AR07^/+* sex comb phenotype.
(48 KB DOC)

Table S4 Two-Way Analysis of Variance of the Effect of Genotype and Temperature on Sex Comb Teeth Number on the Second Tarsal Segment of the First Leg (27 KB DOC)

## Abbreviations

DDC: dopa decarboxylase
TH: tyrosine hydroxylase
