# Supplementary material for: Phenotypic Plasticity in Drosophila Pigmentation Caused by Temperature Sensitivity of a Chromatin Regulator Network
Source: PLoS Genet. 2007 Feb 16;3(2):e30. doi: 10.1371/journal.pgen.0030030 (PMC1797818; doi:10.1371/journal.pgen.0030030)
Supplement: Supplementary file 1 [file pgen.0030030.sd001.doc]

Consensus model of pigment synthesis pathway

A simplified description on the pigment synthesis pathway is included in Figure 8. We have represented only pigments, thus the unpigmented NADA sclerotin which is synthesized from dopamine and plays a major role in cuticle sclerotisation [1,2] is not in the figure. We show a consensus between the models proposed by different authors [1-4]. The main differences between these models are the role of *yellow (y)* and the nature of the black melanin. In *y* mutants, the black melanin disappears and only brown melanin remains, which shows that *y* is required for the production of black melanin [2,4]. In *dopa decarboxylase* *(ddc)* thermosensitive mutants most pigment disappear and a light grey pigment remains, which has been identified as DOPA-melanin because no Dopamine-melanin can be produced without DDC [2,4]. This grey melanin is often considered as a minor pigment in wild type flies, because the amount of black melanin in wild type flies is much higher [2]. This means that most of the black melanin is in fact Dopamine-melanin [2]. Furthermore, it was shown that it is possible to induce in vitro premature cuticle melanisation by incubation of abdominal cuticle with dopamine but not DOPA, *i. e.* through the production of Dopamine-melanin [2]. Interestingly, this premature melanisation is reduced in *y* mutants, which shows that *yellow* is required for the production of dopamine melanin [2]. This suggests that the black melanin which disappears in *y* mutants is, at least partly, Dopamine-melanin [2]. In contrast, other authors consider the DOPA-melanin as the only black melanin present in *Drosophila* and the dopamine melanin as being only the brown dopamine [3,4]. They base this hypothesis on the fact that a mosquito protein related to Yellow, catalyses the conversion of Dopa-chrome, a reaction necessary for the conversion of Dopa to Dopa-melanin [5]. Indeed in order to produce melanin, DOPA and Dopamine are first metabolised into Dopa-chrome and Dopamine-chrome respectively. However, the activity of recombinant *Drosophila* Yellow, renamed Yellow-y, has been tested on Dopa-chrome and instead of being strong as expected it was found to be absent [6]. In contrast, the activity of the related Yellow-f and Yellow-f2 on Dopa-chrome is very high [6]. Crude protein extracts from *y* mutants have a relatively low Dopa-chrome converting enzyme activity compared to wild type, which suggests that Yellow itself does not have this activity but may modulate the activity of Y-f and Y-f2 [6]. In addition, Yellow-f and Yellow-f2 can also use Dopamine-chrome as a substrate [6]. The activity of Yellow-y has not been tested on Dopamine-chrome [6]. So Yellow seems to be involved in the production of Black Dopamine melanin as proposed by Walter and colleagues [2]. We thus present a consensus model, where Yellow catalyses the production of both black/grey DOPA-melanin and black Dopamine-melanin. It explains the antagonist roles on melanin production of *ebony* and *yellow* [4]. Indeed, this antagonism is very strong as the gain of function of yellow does not lead to increased melanin production unless *ebony* is down regulated [4]. Previously, these antagonist roles were explained by product inhibition of the DDC by the excess of dopamine in *ebony* mutants [3].

1. Wittkopp PJ, Carroll SB, Kopp A (2003) Evolution in black and white: genetic control of pigment patterns in Drosophila. Trends Genet 19: 495-504.

2. Walter MF, Zeineh LL, Black BC, McIvor WE, Wright TR, et al. (1996) Catecholamine metabolism and in vitro induction of premature cuticle melanization in wild type and pigmentation mutants of Drosophila melanogaster. Arch Insect Biochem Physiol 31: 219-233.

3. True JR, Yeh SD, Hovemann BT, Kemme T, Meinertzhagen IA, et al. (2005) Drosophila tan Encodes a Novel Hydrolase Required in Pigmentation and Vision. PLoS Genet 1: e63.

4. Wittkopp PJ, True JR, Carroll SB (2002) Reciprocal functions of the Drosophila yellow and ebony proteins in the development and evolution of pigment patterns. Development 129: 1849-1858.

5. Johnson JK, Li J, Christensen BM (2001) Cloning and characterization of a dopachrome conversion enzyme from the yellow fever mosquito, Aedes aegypti. Insect Biochem Mol Biol 31: 1125-1135.

6. Han Q, Fang J, Ding H, Johnson JK, Christensen BM, et al. (2002) Identification of Drosophila melanogaster yellow-f and yellow-f2 proteins as dopachrome-conversion enzymes. Biochem J 368: 333-340.
