# Supplementary material for: Phenotypic Plasticity in Drosophila Pigmentation Caused by Temperature Sensitivity of a Chromatin Regulator Network
Source: PLoS Genet. 2007 Feb 16;3(2):e30. doi: 10.1371/journal.pgen.0030030 (PMC1797818; doi:10.1371/journal.pgen.0030030)
Supplement: Supplementary file 2 [file pgen.0030030.st001.doc]

Table S1

|  | **20°C** | **25°C** | **29°C** |
| --- | --- | --- | --- |
| *bab^AR07^/+* | 0.00 (± 0.00) N=21 | 0.18 (± 0.05) N=36 | 1.56 (± 0.25) N=15 |
| *bab^AR07^/corto^420^* | 0.50 (± 0.09) N=22 | 1.54 (± 0.21) N=13 | 2.64 (± 0.18) N=14 |
| *bab^ARO7^/corto^L1^* | 0.36 (± 0.11) N=14 | 1.42 (± 0.14) N=33 | 2.93 (± 0.15) N=15 |
| *bab^AR07^/corto^7128^* | 0.14 (± 0.07) N=11 | 0.46 (± 0.13) N=12 | 2.30 (± 0.13) N=10 |
| *lolal^k02512^/+;bab^AR07^/+* | 0.25 (± 0.10) N=16 | 1.11 (± 0.17) N=13 | 2.23 (± 0.17) N=15 |
| *bab^AR07^/Trl^13c^* | 0.70 (± 0.11) N=10 | 1.71 (± 0.18) N=14 | 2.72 (± 0.09) N=9 |
| *bab^AR07^/hsp83^e6D^* | 0.40 (± 0.12) N=10 | 0.67 (± 0.10) N=23 | 1.21 (± 0.13) N=19 |
