# Supplementary material for: Phenotypic Plasticity in Drosophila Pigmentation Caused by Temperature Sensitivity of a Chromatin Regulator Network
Source: PLoS Genet. 2007 Feb 16;3(2):e30. doi: 10.1371/journal.pgen.0030030 (PMC1797818; doi:10.1371/journal.pgen.0030030)
Supplement: Supplementary file 3 [file pgen.0030030.st002.doc]

Table S2

|  | df | MS | F | p | Partial Eta Squared |
| --- | --- | --- | --- | --- | --- |
| Corrected model | 20 | 2.973 | 39.053 | <0.001 | .707 |
| Temperature | 2 | 18.201 | 239.090 | <0.001 | .596 |
| Genotype | 6 | 2.254 | 29.606 | <0.001 | .354 |
| Temperature*Genotype | 12 | 0.306 | 4.019 | <0.001 | .130 |
| Error | 324 | 0.076 |  | | |
| Total | 345 |  |  |  |  |
