# Supplementary material for: Phenotypic Plasticity in Drosophila Pigmentation Caused by Temperature Sensitivity of a Chromatin Regulator Network
Source: PLoS Genet. 2007 Feb 16;3(2):e30. doi: 10.1371/journal.pgen.0030030 (PMC1797818; doi:10.1371/journal.pgen.0030030)
Supplement: Supplementary file 4 [file pgen.0030030.st003.doc]

| Source | Dependent Variable | df | Mean Square | F | p | Partial Eta Squared |
| --- | --- | --- | --- | --- | --- | --- |
| Corrected Model | A6L  A6M  A6D  A7L  A7DV | 32  32  32  32  32 | 19.580  18.801  2.453  26.211  18.667 | 214.635  169.862  70.912  315.891  200.191 | <0.001  <0.001  <0.001  <0.001  <0.001 | .959  .948  .884  .971  .956 |
| Temperature | A6L  A6M  A6D  A7L  A7DV | 2  2  2  2  2 | 8.048  16.836  1.135  16.112  13.161 | 88.226  152.107  32.793  194.182  141.145 | <0.001  <0.001  <0.001  <0.001  <0.001 | .373  .506  .181  .567  .487 |
| *bab* | A6L  A6M  A6D  A7L  A7DV | 1  1  1  1  1 | 9.009  1.882  .000  .284  .357 | 98.760  16.999  .000  3.418  3.823 | <0.001  <0.001  1.000  .065  .051 | .250  .054  .000  .011  .013 |
| *corto* | A6L  A6M  A6D  A7L  A7DV | 1  1  1  1  1 | 5.526  37.969  12.675  10.651  4.033 | 60.570  343.042  366.372  128.357  43.255 | <0.001  <0.001  <0.001  <0.001  <0.001 | .169  .536  .552  .302  .127 |
| *Hsp83* | A6L  A6M  A6D  A7L  A7DV | 1  1  1  1  1 | 3.852  24.980  12.675  17.633  15.951 | 42.226  225.688  366.372  212.512  171.060 | <0.001  <0.001  <0.001  <0.001  <0.001 | .124  .432  .552  .417  .365 |
| *crm* | A6L  A6M  A6D  A7L  A7DV | 1  1  1  1  1 | 8.138  7.252  .000  14.008  1.463 | 89.208  65.521  .000  168.825  15.690 | <0.001  <0.001  1.000  <0.001  <0.001 | .231  .181  .000  .362  .050 |
| *Abd-B* | A6L  A6M  A6D  A7L  A7DV | 2  2  2  2  2 | 56.775  1.261  .025  18.311  6.604 | 622.364  11.396  .723  220.681  70.828 | <0.001  <0.001  .486  <0.001  <0.001 | .807  .071  .005  .598  .323 |
| Temperature  **bab* | A6L  A6M  A6D  A7L  A7DV | 2  2  2  2  2 | 7.665  .221  .000  .620  1.815 | 84.022  1.993  .000  7.466  19.464 | <0.001  .138  1.000  <0.001  <0.001 | .361  .013  .000  .048  .116 |
| Temperature  **corto* | A6L  A6M  A6D  A7L  A7DV | 2  2  2  2  2 | 1.113  5.280  4.069  2.729  2.977 | 12.201  47.701  117.608  32.885  31.932 | <0.001  <0.001  <0.001  <0.001  <0.001 | .076  .243  .442  .181  .177 |
| Temperature  **Hsp83* | A6L  A6M  A6D  A7L  A7DV | 2  2  2  2  2 | .757  1.544  4.069  4.408  8.132 | 8.296  13.948  117.608  53.128  87.209 | <0.001  <0.001  <0.001  <0.001  .000 | .053  .086  .442  .263  .370 |
| Temperature  **crm* | A6L  A6M  A6D  A7L  A7DV | 2  2  2  2  2 | 4.269  .160  .000  10.308  .454 | 46.799  1.445  .000  124.233  4.865 | <0.001  .237  1.000  . <0.001  .008 | .240  .010  .000  .456  .032 |
| Temperature  **Abd-B* | A6L  A6M  A6D  A7L  A7DV | 4  4  4  4  4 | 4.500  4.409  .025  4.753  2.681 | 49.329  39.835  .723  57.279  28.757 | <0.001  <0.001  .577  <0.001  <0.001 | .399  .349  .010  .435  .279 |
| *bab***corto* | A6L  A6M  A6D  A7L  A7DV | 1  1  1  1  1 | .752  8.802  .000  1.251  .264 | 8.244  79.526  .000  15.071  2.828 | .004  <0.001  1.000  <0.001  .094 | .027  .211  .000  .048  .009 |
| *bab***Hsp83* | A6L  A6M  A6D  A7L  A7DV | 1  1  1  1  1 | .117  5.963  .000  .102  .000 | 1.285  53.875  .000  1.230  .001 | .258  <0.001  1.000  .268  .970 | .004  .154  .000  .004  .000 |
| *bab***crm* | A6L  A6M  A6D  A7L  A7DV | 1  1  1  1  1 | .876  2.700  .000  .408  .501 | 9.597  24.394  .000  4.921  5.368 | .002  <0.001  1.000  .027  .021 | .031  .076  .000  .016  .018 |
| *corto***Hsp83* | A6L  A6M  A6D  A7L  A7DV | 1  1  1  1  1 | .188  1.875  12.675  4.602  2.969 | 2.061  16.940  366.372  55.463  31.840 | .152  <0.001  <0.001  <0.001  <0.001 | .007  .054  .552  .157  .097 |
| Temperature  **bab***corto* | A6L  A6M  A6D  A7L  A7DV | 2  2  2  2  2 | .882  2.616  .000  .907  2.473 | 9.666  23.636  .000  10.928  26.522 | <0.001  <0.001  1.000  <0.001  <0.001 | .061  .137  .000  .069  .152 |
| Temperature  **bab***Hsp83* | A6L  A6M  A6D  A7L  A7DV | 2  2  2  2  2 | .058  .640  .000  .182  .845 | .634  5.779  .000  2.191  9.059 | .531  .003  1.000  .114  <0.001 | .004  .037  .000  .015  .057 |
| Temperature  **bab***crm* | A6L  A6M  A6D  A7L  A7DV | 2  2  2  2  2 | 1.226  .427  .000  .108  1.676 | 13.434  3.854  .000  1.306  17.969 | <0.001  .022  1.000  .273  <0.001 | .083  .025  .000  .009  .108 |
| Temperature  **corto***Hsp83* | A6L  A6M  A6D  A7L  A7DV | 2  2  2  2  2 | .990  .919  4.069  7.157  1.448 | 10.848  8.301  117.608  86.252  15.527 | <0.001  <0.001  <0.001  <0.001  <0.001 | .068  .053  .442  .367  .095 |
| Error | A6L  A6M  A6D  A7L  A7DV | 297  297  297  297  297 | .091  .111  .035  .083  .093 |  | | |
| Total | A6L  A6M  A6D  A7L  A7DV | 330  330  330  330  330 |  | | | |
