# Supplementary material for: Phenotypic Plasticity in Drosophila Pigmentation Caused by Temperature Sensitivity of a Chromatin Regulator Network
Source: PLoS Genet. 2007 Feb 16;3(2):e30. doi: 10.1371/journal.pgen.0030030 (PMC1797818; doi:10.1371/journal.pgen.0030030)
Supplement: Supplementary file 5 [file pgen.0030030.st004.doc]

| Source | Dependent variable | df | Mean Square | F | p | Partial Eta Squared |
| --- | --- | --- | --- | --- | --- | --- |
| Corrected  Model | A6L  A6M  A6D  A7L  A7DV | 5  5  5  5  5 | 21.772  23.147  .000  32.916  26.794 | 202.266  151.505  .  537.608  202.266 | <0.001  <0.001  .  <0.001  <0.001 | .949  .933  .  .980  .959 |
| Temperature | A6L  A6M  A6D  A7L  A7DV | 2  2  2  2  2 | 51.782  47.454  .000  82.270  64.845 | 481.074  310.609  .  1343.688  612.305 | <0.001  <0.001  .  <0.001  <0.001 | .947  .920  .  .980  .958 |
| *bab* | A6L  A6M  A6D  A7L  A7DV | 1  1  1  1  1 | 2.204  13.067  .000  .001  .551 | 20.477  85.527  .  .017  5.203 | <0.001  <0.001  .  .897  .027 | .275  .613  .  .000  .088 |
| Temperature  **bab* | A6L  A6M  A6D  A7L  A7DV | 2  2  2  2  2 | 1.545  3.879  .000  .020  1.864 | 14.352  25.391  .  .323  17.597 | <0.001  <0.001  .  .725  <0.001 | .347  .485  .  .012  .395 |
| Error | A6L  A6M  A6D  A7L  A7DV | 54  54  54  54  54 | .108  .153  .000  .061  .106 | 481.074  310.609  .  1343.688  612.305 | <0.001  <0.001  .  <0.001  <0.001 | .947  .920  .  .980  .958 |
| Total | A6L  A6M  A6D  A7L  A7DV | 60  60  60  60  60 |  |  |  |  |
